# Degradation Performance and Mechanism of Methyl Orange by Iron-Modified Sludge Biochar-Activated Peroxymonosulfate

**DOI:** 10.3390/nano16191233

**Published:** 2026-09-30

**Authors:** Meng-Yuan Li, Jiang-Li Yu, Mei-Qi Ren, Tao Hou, Dan Cui

**Affiliations:** National Engineering Laboratory for Advanced Municipal Wastewater Treatment and Reuse Technology, College of Environmental Science and Engineering, Beijing University of Technology, Beijing 100124, China; s202583083@emails.bjut.edu.cn (M.-Y.L.); 18501312951@163.com (J.-L.Y.); renmeiqi2023@emails.bjut.edu.cn (M.-Q.R.); ht001114@163.com (T.H.)

**Keywords:** azo dye, sludge biochar, iron modification, peroxymonosulfate, singlet oxygen, degradation mechanism

## Abstract

Azo dye wastewater poses a persistent environmental challenge because of its high toxicity, poor biodegradability, and widespread occurrence in textile and dyeing effluents, highlighting the need for efficient and sustainable treatment technologies. In this study, iron-modified sludge biochar (Fe@SBC) was prepared via impregnation–pyrolysis and employed to activate peroxymonosulfate (PMS) for methyl orange (MO) degradation, providing a dual benefit of sludge valorization and pollutant removal. Iron modification increased the specific surface area from 50.93 to 66.54 m^2^·g^−1^ and total pore volume from 0.085 to 0.309 cm^3^·g^−1^, while introducing Fe_3_O_4_, Fe^2+^/Fe^3+^ redox pairs, and oxygen-containing functional groups that significantly enhanced electron transfer. The Fe@SBC/PMS system achieved 95.2 ± 0.2% MO removal within 25 min under optimal conditions, notably outperforming unmodified biochar (86.0 ± 0.6%). The catalyst exhibited excellent pH adaptability (3–11) and maintained 88.0 ± 0.8% removal after five cycles. Quenching experiments and EPR analysis identified singlet oxygen (^1^O_2_) as the dominant reactive species (~80% contribution), with sulfate and hydroxyl radicals playing auxiliary roles. LC-MS analysis proposed three parallel degradation pathways involving azo bond cleavage, hydroxylation, and desulfonation. These findings demonstrate that Fe@SBC is an effective PMS activator and provide mechanistic insight into the design of sludge-derived carbon catalysts for advanced oxidation processes, offering a promising approach for simultaneous sludge resource recovery and azo dye wastewater treatment.

## 1. Introduction

Azo dyes are among the most widely used synthetic colorants and constitute a major class of refractory pollutants in industrial effluents, especially from textile, printing, and dyeing industries. Owing to their aromatic structures and stable azo bonds (–N=N–), these compounds are highly resistant to biodegradation and can persist in aquatic environments for prolonged periods [1,2]. It is estimated that more than 700,000 tons of synthetic dyes are produced annually worldwide, of which azo dyes account for over 60% [3]. Their concentrations in industrial wastewater commonly range from tens to hundreds of mg·L^−1^ and may reach even higher levels in untreated dyeing effluents [4]. In addition to aesthetic deterioration of water bodies, azo dyes and their transformation products, particularly aromatic amines, pose significant ecotoxicological and health risks [5]. Therefore, the development of efficient and sustainable technologies for azo dye removal remains an important environmental challenge.

A variety of treatment methods have been applied for dye-containing wastewater, including biological treatment, adsorption, membrane separation, coagulation/flocculation, and advanced oxidation processes (AOPs). Biological processes are economical but often show slow degradation kinetics and incomplete mineralization for recalcitrant dyes [5]. Adsorption and membrane filtration can effectively remove color, yet they mainly transfer pollutants to another phase and generate secondary wastes [6]. Coagulation/flocculation is simple and effective for some dye classes, but its efficiency is limited for soluble dyes, and it usually produces large amounts of sludge [7]. Among AOPs, Fenton oxidation, ozonation, and photocatalysis have shown good oxidation potential. However, their practical application is often constrained by narrow pH requirements, sludge generation, low mineralization efficiency, by-product formation, or poor energy utilization [2,8]. These limitations have stimulated increasing interest in sulfate radical-based advanced oxidation processes (SR-AOPs) [9,10], particularly peroxymonosulfate (PMS)-based systems, because of their strong oxidation ability, relatively wide pH adaptability, and potential to generate both radical and non-radical reactive oxygen species [11,12,13].

The performance of PMS-based oxidation largely depends on the catalyst used for PMS activation. In this regard, biochar has attracted extensive attention as a low-cost and tunable carbonaceous catalyst due to its porous structure, defect-rich carbon framework, and abundant surface functional groups [14,15]. Most previous studies have focused on plant-derived biochars, where PMS activation is mainly associated with graphitic domains, oxygen-containing groups, and structural defects [16,17]. In contrast, sludge-derived biochar provides a distinct catalytic platform because municipal sludge inherently contains mineral components and endogenous metals such as Fe, Al, and Ca [18]. During pyrolysis, these components can interact with the carbon matrix to form metal–carbon interfacial sites, endowing sludge biochar with dual functionality in pollutant adsorption and oxidant activation [19]. From a resource recovery perspective, converting excess sludge into catalytic biochar also offers an attractive route for simultaneous waste valorization and wastewater treatment.

Among sludge-derived catalysts, iron-modified sludge biochar (Fe@SBC) has shown particular promise for PMS activation. Fe incorporation can markedly enhance catalytic performance by increasing the density of accessible active sites, promoting Fe^2+^/Fe^3+^ redox cycling, and facilitating electron transfer at Fe–O–C interfacial structures [20,21]. In addition, Fe species may regulate pore development during pyrolysis and favor the generation of defect sites and oxygen-containing groups, thereby improving both adsorption capacity and PMS activation efficiency [22]. Recent studies have demonstrated that Fe-modified sludge biochars can efficiently remove azo dyes and other refractory organics through the combined action of radical species (SO_4_^•−^ and ^•^OH) and non-radical pathways, particularly singlet oxygen (^1^O_2_) [23,24,25,26,27]. These findings suggest that Fe@SBC is not merely an adsorbent or conventional Fenton-like catalyst, but a multifunctional PMS activator with considerable potential for practical wastewater treatment.

Nevertheless, several critical issues remain unresolved. First, the effects of pyrolysis conditions and Fe incorporation on the structural evolution of sludge biochar, including pore development, Fe speciation, and surface chemistry, have not been systematically clarified. Second, although both radical and non-radical pathways have been reported in sludge biochar/PMS systems, their relative roles and mechanistic interplay remain poorly understood. Third, many existing studies emphasize decolorization efficiency, while the mineralization behavior and transformation pathways of azo dyes receive much less attention. Therefore, a systematic investigation linking catalyst preparation, material structure, PMS activation behavior, and pollutant degradation pathways is still needed.

To fill these gaps, the main goal of this study is to develop a high-performance iron-modified sludge biochar catalyst for PMS activation and to comprehensively elucidate the structure–activity relationships and degradation mechanisms governing the removal of azo dyes. Accordingly, the present study employs municipal excess sludge as a precursor to synthesize Fe@SBC via an impregnation–pyrolysis strategy and systematically investigates its catalytic performance in PMS activation for the degradation of methyl orange (MO). The specific objectives are: (i) to optimize the pyrolysis and modification conditions for constructing Fe@SBC with enhanced catalytic activity; (ii) to identify the structural evolution, iron speciation, pore characteristics, and surface chemistry responsible for catalyst activation; (iii) to evaluate the effects of operating parameters, environmental matrix components, and cycling use on MO degradation; and (iv) to elucidate the PMS activation mechanism by resolving the roles of radical and non-radical pathways, alongside the degradation and mineralization behaviors of MO. This study provides mechanistic insights for the rational design of Fe-modified sludge-derived carbon catalysts and offers a viable technological strategy for integrating sludge resource recovery with the efficient treatment of dye-laden wastewater.

## 2. Materials and Methods

### 2.1. Chemicals

All chemicals were used as received without additional purification, and the detailed supplier and purity information of all reagents is provided in Text S1.

### 2.2. Preparation of SBC and Fe@SBC

The sludge was collected from the secondary sedimentation tank of Gaobeidian Wastewater Treatment Plant (Beijing, China). The raw sludge was first centrifugally dewatered; the obtained wet sludge cake (solid concentration ca. 200 g·L^−1^, as measured by the mass of the dewatered sludge per liter) was used as the precursor for biochar preparation. The sludge was repeatedly washed with deionized water to remove soluble impurities, dried at 105 °C, ground, and sieved before use.

Pristine SBC was prepared in a tubular furnace under an N_2_ atmosphere. To investigate the effect of pyrolysis conditions on the physicochemical properties of SBC, two variables were considered: pyrolysis temperature (400, 600, and 800 °C) and heating rate (5, 10, and 15 °C·min^−1^), while the holding time at the target temperature was fixed at 2 h. The optimal preparation conditions were selected based on the resulting surface morphology, pore characteristics, and catalytic performance.

Fe@SBC was then synthesized under the selected optimal pyrolysis conditions using an impregnation–pyrolysis method. Briefly, 3.0 g of dried sludge and 0.3 g of FeCl_3_·6H_2_O were dispersed in 90 mL of deionized water and magnetically stirred at room temperature for 12 h to ensure sufficient impregnation. The mixture was then washed five times with deionized water and absolute ethanol, dried, ground uniformly, and pyrolyzed at 800 °C for 2 h under N_2_ protection to obtain Fe@SBC. For comparison, the unmodified sludge precursor was pyrolyzed under the same conditions to obtain SBC. The preparation flow chart is shown in Figure S1.

### 2.3. Material Characterization Methods

#### 2.3.1. SEM-EDS Analysis

SEM imaging was conducted on field-emission scanning electron microscopes (FE-SEM). SBC was characterized with a Hitachi SU8600 FE-SEM (Hitachi High-Tech Corporation, Tokyo, Japan) at 15.0 kV to reveal the overall porous structure, whereas Fe@SBC was characterized with a high-resolution Hitachi SU9000 ultra-high resolution FE-SEM (Hitachi High-Tech Corporation, Tokyo, Japan) at 3.0 kV to better resolve the surface iron species and avoid surface charging and electron-beam damage. All samples were dried, mounted on conductive tape, and sputter-coated with gold prior to observation.

#### 2.3.2. XPS Analysis

X-ray photoelectron spectroscopy (XPS, ESCALAB 250Xi, Thermo Fisher Scientific Inc., Waltham, MA, USA) was employed to analyze the elemental composition and chemical states of the samples.

#### 2.3.3. XRD Analysis

X-ray diffraction (XRD) was used to analyze crystal structures with Cu Kα radiation (λ = 0.15406 nm) at a scanning rate of 5° min^−1^. Two instruments were employed: a Rigaku SmartLab multipurpose X-ray diffractometer (Rigaku Corporation, Tokyo, Japan) for rapid screening of biochar samples under different pyrolysis conditions (2θ = 20–80°), and a Bruker D8 Advance X-ray diffractometer (Bruker AXS GmbH, Karlsruhe, Germany) for fine crystal structure characterization of optimized SBC and Fe@SBC (2θ = 5–90°). The core test parameters of the two instruments are consistent, and the XRD results are comparable.

#### 2.3.4. BET Surface Area and Pore Structure Analysis

The specific surface area and pore structure were measured using an N_2_ adsorption–desorption analyzer (ASAP 2020HD88, Micromeritics Instrument Corporation, Norcross, GA, USA). Prior to analysis, samples were degassed to remove surface-adsorbed impurities. The Brunauer–Emmett–Teller (BET) method was used to calculate the specific surface area from the adsorption isotherms.

#### 2.3.5. Electrochemical Analysis

Cyclic voltammetry (CV) measurements were performed on an electrochemical workstation (CHI1000C, CH Instruments, Shanghai, China) to evaluate the electrochemical properties of biochar samples. A three-electrode system was adopted with biochar-coated glassy carbon as the working electrode, platinum plate as the counter electrode, and Ag/AgCl as the reference electrode. 0.1 mol/L Na_2_SO_4_ aqueous solution was used as the supporting electrolyte. The scanning potential range was set from −0.33 V to 0.6 V (vs. Ag/AgCl), and the scan rate was 0.05 V/s.

### 2.4. Chemical Analysis and Calculation Methods

The concentration of MO was determined by UV–vis spectrophotometry (UV1600, Optosky (Xiamen) Photonics Inc., Xiamen, China) at 464 nm. A standard concentration gradient (0–10 mg·L^−1^) was prepared to draw a standard calibration curve (R^2^ > 0.999). After sampling, 1 mL water sample was filtered through a 0.22 μm PES membrane, mixed with 0.5 mL methanol to instantaneously terminate the oxidation reaction before the absorbance test.

Total organic carbon (TOC) was measured using a TOC analyzer, and chemical oxygen demand (COD) was determined by the dichromate method. Before analysis, all samples were filtered through 0.45 μm membranes to remove suspended solids and diluted when necessary to ensure that the absorbance fell within the instrument’s linear range [9,28]. Inorganic carbon was eliminated by acidification before testing, and TOC removal rate was calculated by comparing TOC_0_ (initial) and T_t_ (residual) at a set reaction time. COD value was determined by the standard potassium dichromate reflux digestion method. Digestion temperature was 165 °C with 2 h of constant heating, and absorbance was measured at 600 nm for quantitative calculation.

The pseudo-first-order kinetic model was used to describe the kinetic behavior of adsorption and pollutant degradation processes. The differential equation of the pseudo-first-order model is expressed as Equation (1):

(1) $$\frac{dq_{t}}{dt}=k_{1}\left(q_{e}-q_{t}\right)$$

After integration and rearrangement under the boundary conditions of *t* = 0, *q*_t_ = 0 and *t* = *t*, *q*_t_ = *q*_t_, the linear form can be obtained as Equation (2):

(2) $$\ln\left(q_{e}-q_{t}\right)=\ln q_{e}-k_{1}t$$

where *q*_e_ (mg·g^−1^) and *q*_t_ (mg·g^−1^) represent the adsorption capacity at equilibrium and at time *t*; *k*_1_ (min^−1^) is the rate constant of the pseudo-first-order model.

For pollutant degradation experiments, the model can be further simplified based on concentration variation:

(3) $$\ln\frac{C_{0}}{C_{t}}=k_{\mathrm{obs}}t$$

where *C*_0_ and *C*_t_ are the initial and residual pollutant concentrations at time *t*; *k*_obs_ is the apparent pseudo-first-order rate constant for MO degradation.

### 2.5. Radical Identification by EPR

Electron paramagnetic resonance (EPR, BRUKER EMXPLUS, Karlsruhe, Germany) was used to qualitatively identify the reactive oxygen species generated in the system. DMPO was used as the spin-trapping agent for ^•^OH and SO_4_^•−^, while TEMP was used to detect ^1^O_2_. The formation of DMPO–OH, DMPO–SO_4_^−^, and TEMPO adducts was identified by their characteristic EPR signals. The detailed instrument parameters, spin-trapping agents and test procedures are provided in Text S2.

### 2.6. Identification of Degradation Intermediates

High-resolution LC-QTOF-MS was adopted to separate and identify organic intermediates generated during MO degradation, and detailed chromatographic and mass spectrometric operating parameters are summarized in Text S3.

### 2.7. Quenching Experiments

To probe the contribution of different reactive species, quenching experiments were performed using methanol (MeOH) and tert-butanol (TBA) as scavengers for SO_4_^•−^and ^•^OH, p-benzoquinone (BQ) as a scavenger for O_2_^•−^, and L-histidine as a scavenger for ^1^O_2_. The degradation efficiencies before and after addition of each quencher were compared under otherwise identical conditions.

### 2.8. Experimental Design

Unless otherwise stated, the initial concentration of the MO solution was 20 mg·L^−1^. All experiments were conducted in a constant-temperature water-bath shaker at 25 °C and 200 rpm. Unless otherwise stated, the initial pH was 8.2. Fe@SBC was first mixed with MO solution for 30 min to achieve adsorption equilibrium. PMS was then added to initiate the oxidation reaction. At predetermined intervals (0, 5, 10, 15, 20, and 25 min after PMS addition), 1 mL aliquots were withdrawn, filtered through a 0.22 μm polyethersulfone membrane, and immediately quenched with 0.5 mL methanol prior to UV–vis analysis. A full range of single-factor variables, including catalyst dosage, PMS concentration, initial pH and coexisting anions, were investigated, with complete fixed and variable reaction parameters summarized in Table S1.

### 2.9. Statistical Analysis

All experiments were performed in triplicate, and the results are expressed as mean ± standard deviation (SD). Statistical significance of the performance difference between SBC/PMS and Fe@SBC/PMS systems was evaluated using an independent two-sample t-test, with *p* < 0.05 considered statistically significant.

## 3. Results and Discussion

### 3.1. Optimization of Preparation Conditions for SBC

#### 3.1.1. Effects of Pyrolysis Temperature and Heating Rate on the Morphology and Structure of SBC

**Figure 1:**
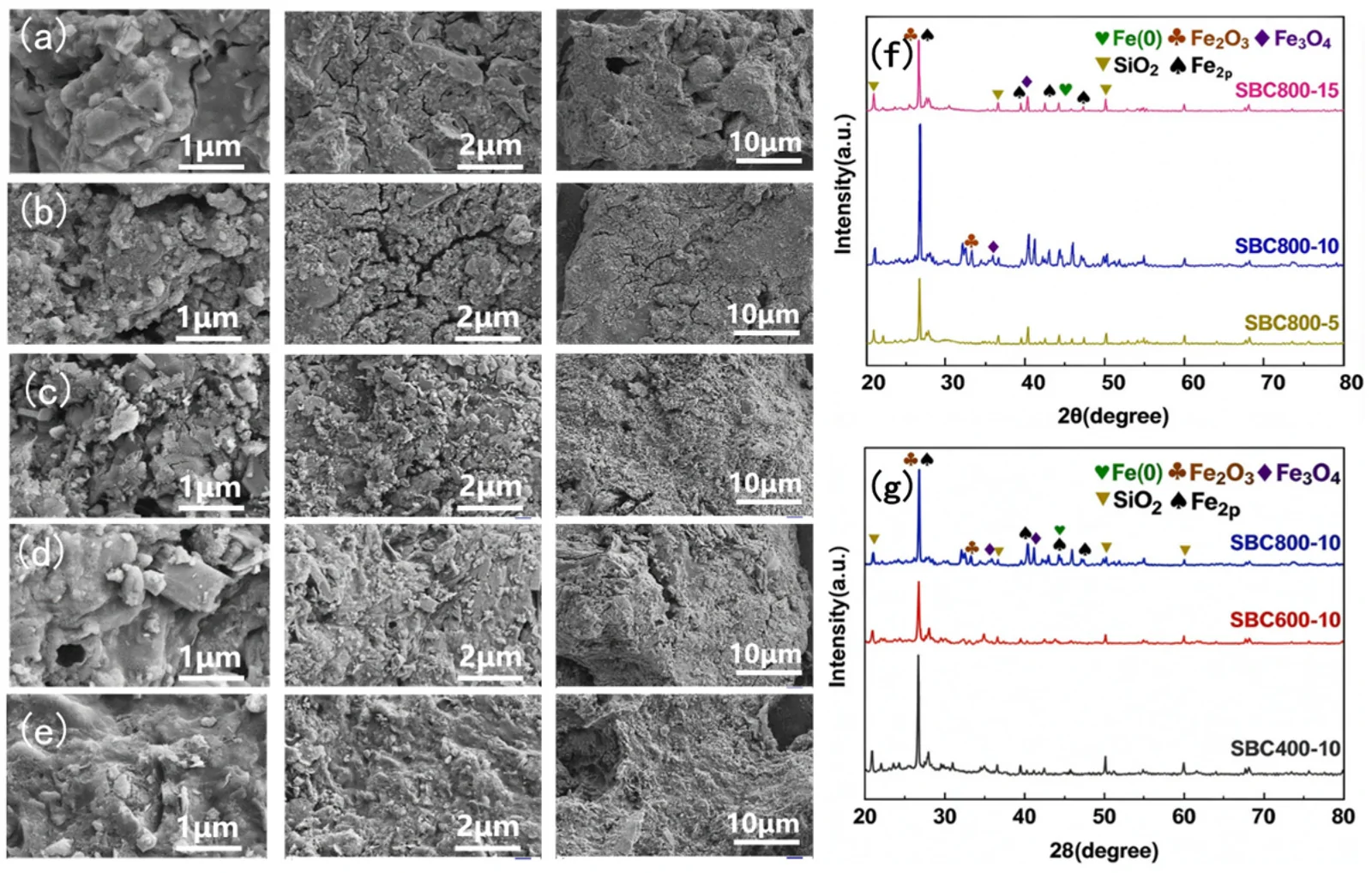
SEM images of sludge biochar under different pyrolysis conditions: (**a**) 400-10, (**b**) 600-10, (**c**) 800-10, (**d**) 800-5, (**e**) 800-15. SEM images were acquired on a Hitachi SU8600 FE-SEM (Hitachi High-Tech Corporation, Tokyo, Japan) at 15.0 kV. XRD patterns (**f**) at different heating rates and (**g**) at different pyrolysis temperatures were recorded on a Rigaku SmartLab (Rigaku Corporation, Tokyo, Japan) over 2θ = 20–80°.

The morphology and structure of SBC are governed by pyrolysis temperature and heating rate, which jointly determine pore development, carbon skeleton reconstruction, and the transformation of endogenous mineral phases [29]. SEM images (Figure 1a–c) showed that, at a fixed heating rate of 10 °C·min^−1^, the surface morphology of SBC changed markedly as the pyrolysis temperature increased from 400 to 800 °C. SBC prepared at 400 °C exhibited a relatively compact surface with limited visible pores, indicating incomplete decomposition of organic components. At 600 °C, the surface became rougher and isolated pores began to emerge, suggesting progressive devolatilization and partial reconstruction of the carbon matrix. When the temperature increased to 800 °C, an interconnected porous framework with well-defined channels was formed, implying that high-temperature treatment facilitated pore development and improved structural openness. This may be attributable to intensified volatile release and thermal rearrangement of the carbon matrix [30].

At a fixed pyrolysis temperature of 800 °C, the heating rate also significantly influenced the final morphology (Figure 1c–e). At 5 °C·min^−1^, pores were present, but local shrinkage and partial densification could still be observed, likely due to prolonged thermal exposure. Increasing the heating rate to 10 °C·min^−1^ resulted in a more uniformly distributed porous structure with relatively intact pore walls, indicating a better balance between volatile release and framework stabilization. In contrast, at 15 °C·min^−1^, the surface became comparatively smoother, and fewer pores were visible, suggesting that excessively rapid heating may induce premature surface densification that traps volatiles and suppresses internal pore development. These observations indicate that 800 °C and 10 °C·min^−1^ provided the most favorable conditions for generating a more open and accessible porous structure.

The XRD patterns further reflected the structural evolution of SBC under different preparation conditions (Figure 1f,g). All samples showed a broad diffraction feature in the low-angle region, characteristic of poorly ordered carbonaceous structures, together with reflections attributable to mineral phases inherited from the sludge matrix [31]. While diffraction positions remained essentially unchanged, relative intensities varied with temperature and heating rate, indicating alterations in phase crystallinity rather than the formation of new phases. Notably, SBC-800-10 (prepared at 800 °C and 10 °C·min^−1^) displayed a more pronounced amorphous carbon-related peak and discernible reflections in the 30–40° region (tentatively assigned to iron oxides), suggesting that this condition favored the exposure of catalytically relevant mineral domains while preserving defective carbon structures.

#### 3.1.2. Effects of Pyrolysis Conditions on the Surface Chemical Properties of SBC

**Figure 2:**
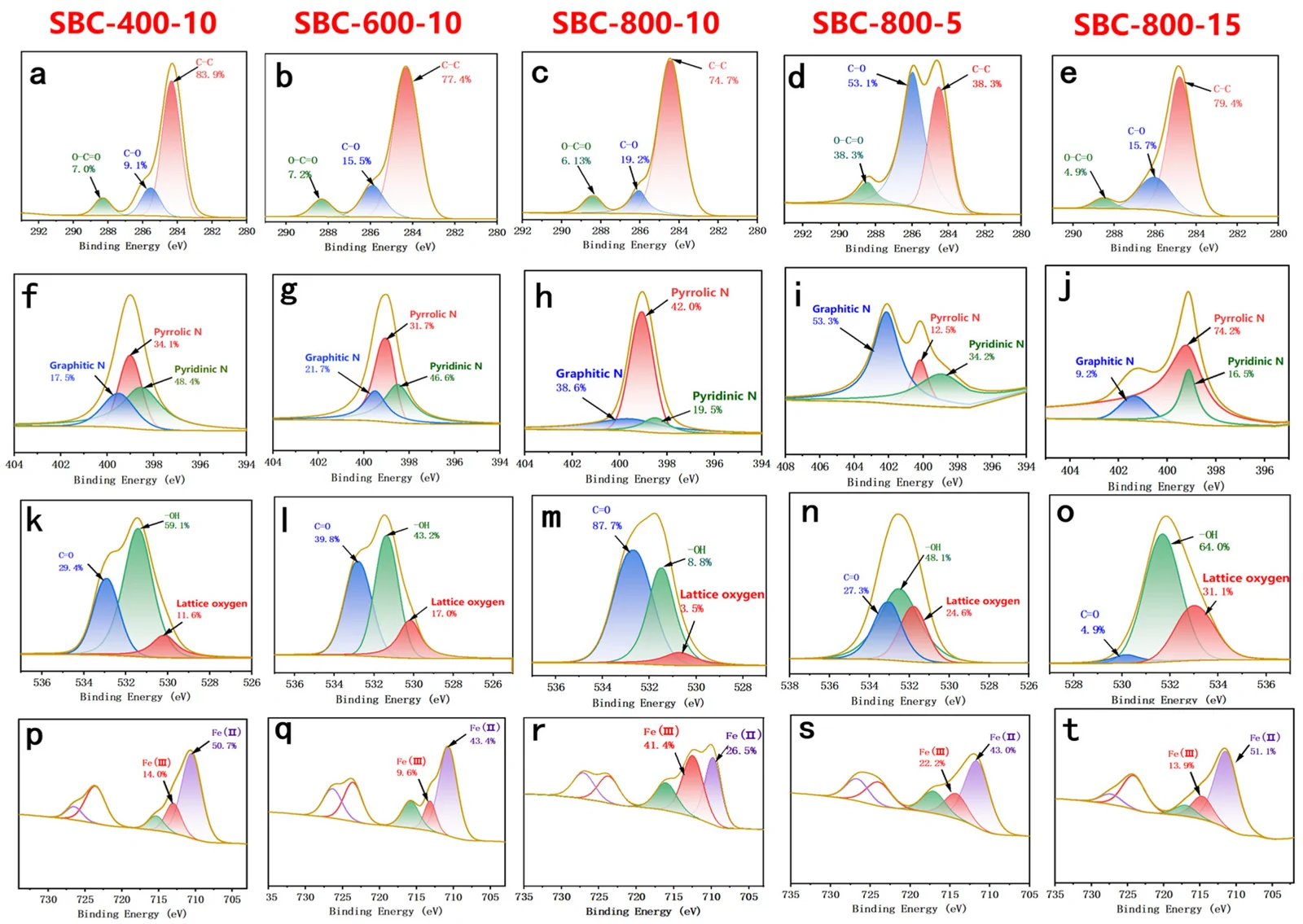
XPS spectra of sludge biochar under different pyrolysis conditions: (**a**–**e**) high-resolution C1s spectra, (**f**–**j**) high-resolution N1s spectra, (**k**–**o**) high-resolution O1s spectra, (**p**–**t**) high-resolution Fe 2p spectra.

XPS was employed to investigate the evolution of surface chemical states of SBC prepared under different pyrolysis conditions. The C 1s spectra could be deconvoluted into C–C/C=C (284.8 eV), C–O (286.2 eV), and O–C=O (288.8 eV) species (Figure 2a–e). At a fixed heating rate of 10 °C·min^−1^, increasing the pyrolysis temperature from 400 to 800 °C resulted in a decrease in the relative fraction of C–C/C=C and an increase in C–O species. Compared with SBC-400-10, SBC-800-10 showed a 9.16% decrease in C–C/C=C and a 10.05% increase in C–O. This trend suggests that high-temperature pyrolysis induced substantial surface reconstruction, possibly accompanied by the exposure of defect sites and oxygen-containing functionalities. Considering the complexity of sludge-derived carbon materials, this change may also reflect the influence of mineral-rich phases on the surface chemical environment.

The N 1s spectra showed a gradual decrease in pyridinic N and an increase in graphitic N with increasing pyrolysis temperature and heating rate (Figure 2f–j). This evolution is consistent with the thermal transformation of less stable nitrogen species into more condensed and thermally stable graphitic N. Since graphitic N is generally associated with improved electronic properties of carbonaceous catalysts, its enrichment may be beneficial for interfacial PMS activation.

In the O 1s spectra, the proportion of lattice oxygen decreased with increasing temperature, whereas the contribution of C=O-related oxygen increased under suitable pyrolysis conditions (Figure 2k–o). Notably, the C=O fraction first increased and then declined with increasing heating rate, indicating that oxygen functionality retention was governed by the competition between thermal decomposition and structural rearrangement. Carbonyl groups have been frequently associated with non-radical PMS activation and ^1^O_2_ generation. Therefore, their enrichment may contribute positively to catalytic performance.

The Fe 2p spectra further indicated that the relative abundance of Fe (III) increased with increasing pyrolysis severity (Figure 2p–t). This result implies that the oxidation state and local coordination environment of endogenous iron species were modified during pyrolysis, which may affect their reactivity toward PMS.

The XPS results show that both temperature and heating rate significantly regulated the surface chemistry of SBC. Among the tested conditions, 800 °C and 10 °C·min^−1^ provided a relatively balanced combination of oxygen-containing groups, nitrogen configuration, and iron valence state, which is consistent with the superior catalytic performance discussed below.

#### 3.1.3. Catalytic Performance of SBC Prepared Under Different Pyrolysis Conditions

**Figure 3:**
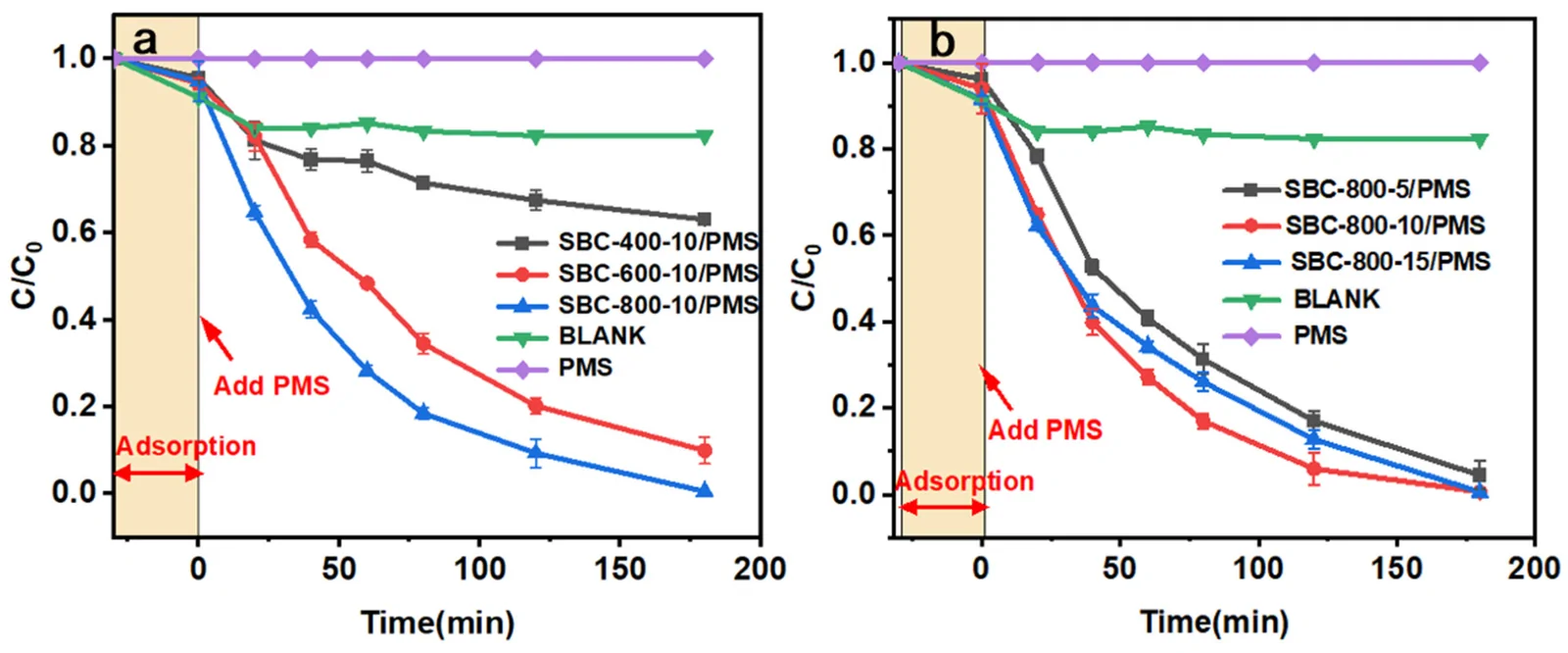
Removal efficiency of pollutants by sludge biochar under different pyrolysis conditions: (**a**) different heating rates, (**b**) different pyrolysis temperatures. Initial MO concentration: 20 mg·L^−1^.

The catalytic activity of SBC prepared under different conditions was evaluated by PMS activation for MO degradation. At a fixed heating rate of 10 °C·min^−1^, the catalytic activity increased with pyrolysis temperature, and SBC-800-10 exhibited the best performance among the tested samples; complete removal can be achieved within 150 min (Figure 3a). This result is consistent with the observed development of pore structure and the evolution of surface chemical properties revealed by SEM, XRD, and XPS.

At a fixed pyrolysis temperature of 800 °C, the catalytic performance depended strongly on the heating rate. SBC prepared at 10 °C·min^−1^ showed the highest activity, with an apparent rate constant of 0.021 ± 0.001 min^−1^ and complete MO removal within 150 min (Figure 3b). Lower activity was observed at both 5 and 15 °C·min^−1^, indicating that either excessive thermal densification or insufficiently controlled pore evolution was detrimental to the formation of accessible active sites. Therefore, 800 °C and 10 °C·min^−1^ were selected as the optimal preparation conditions for SBC and adopted for subsequent iron modification.

### 3.2. Physicochemical Characteristics of Fe@SBC

#### 3.2.1. Surface Morphology and Elemental Distribution

**Figure 4:**
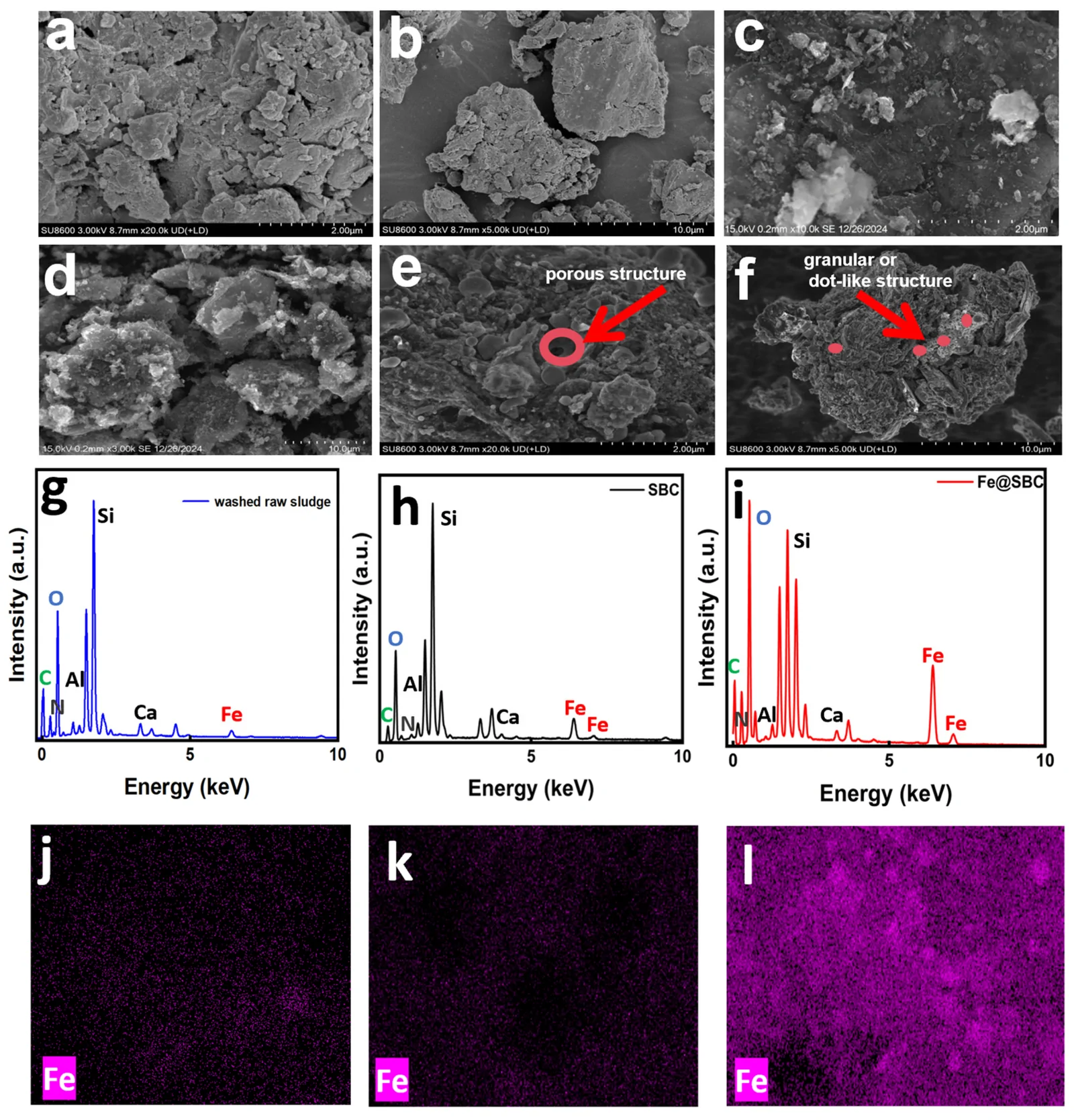
SEM images, EDS spectra and Fe elemental mapping of washed raw sludge ((**a**,**b**,**g**,**j**), 3.0 kV, SU9000), SBC ((**c**,**d**,**h**,**k**), 15.0 kV, SU8600) and Fe@SBC ((**e**,**f**,**i**,**l**), 3.0 kV, SU9000).

SEM images (Figure 4) reveal the morphological evolution from washed raw sludge to SBC and Fe@SBC. The pristine washed raw sludge (Figure 4a,b) shows irregular massive morphology with a dense and smooth surface, where organic components encapsulate mineral particles and limit exposed surface sites. After pyrolysis, SBC (Figure 4c,d) exhibits a rougher and more fragmented surface with developed cracks and primary pores, as organic matter decomposes and volatilizes to expose inherent mineral phases and increase available surface sites. For Fe@SBC (Figure 4e,f), uniformly distributed particulate iron-containing species are observed on the further roughened biochar matrix, and the modification process further optimizes the porous structure. Such features facilitate pollutant diffusion and adsorption, provide abundant catalytic active sites, and promote electron transfer via iron–carbon interactions.

EDS analysis further confirmed the successful incorporation of iron. Both SBC and Fe@SBC were mainly composed of C and O, with minor amounts of Si, Al, and Ca inherited from the sludge matrix (Figure 4g–i). The atomic fractions (at. %) of core elements (C, N, O, Fe) are summarized in Table S2. After pyrolysis, the content increased from 26.69 at. % to 46.92 at. % due to carbon enrichment during organic matter decomposition, while N and O decreased to 2.39 at. % and 47.79 at. % respectively, owing to volatilization of nitrogen- and oxygen-containing organics. The Fe content rose from 1.25 at. % in washed raw sludge to 2.89 at. % in SBC, reflecting the relative enrichment of non-volatile indigenous iron during pyrolysis. After iron modification, Fe@SBC shows a further increase in Fe atomic fraction to 6.34 at. %, accompanied by an increase in O, confirming the successful immobilization of iron oxide species on the biochar matrix. Fe elemental mapping (Figure 4j–l) showed a markedly enhanced and relatively uniform iron distribution after modification, indicating that iron species were well dispersed on the biochar surface. This uniform dispersion is important because it minimizes local agglomeration, increases the density of accessible active centers, and supports efficient PMS activation.

#### 3.2.2. Surface Chemistry, Crystal Structure, and Porosity

**Figure 5:**
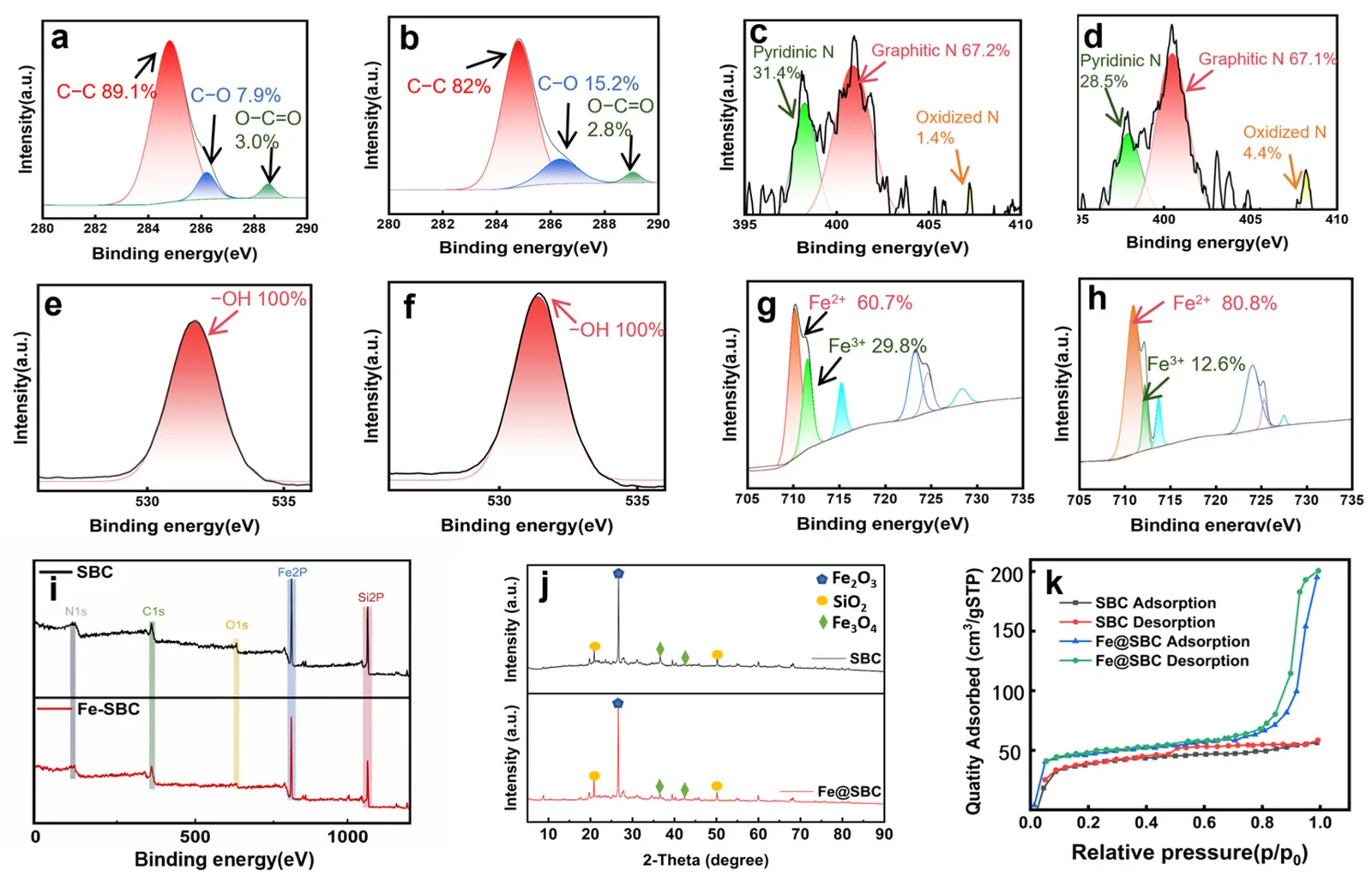
XPS spectra of SBC and Fe@SBC: (**a**,**b**) C 1s, (**c**,**d**) N 1s, (**e**,**f**) O 1s, and (**g**,**h**) Fe 2p; (**i**) XRD patterns recorded on a Bruker D8 Advance (Germany) over 2θ = 5–90°; (**j**) XRD patterns (scanned over 2θ = 5–90°); (**k**) N_2_ adsorption–desorption isotherms.

XPS analysis showed that Fe modification significantly altered the surface chemical environment of SBC. In the C 1s spectra (Figure 5a,b), the proportion of C–C/C=C decreased from 89.1% to 82%, indicating partial disruption of the carbon framework and generation of more defective sites during Fe-assisted pyrolysis. Notably, the proportion of C–O groups nearly doubled from 7.9% to 15.2%, while the content of O–C=O changed only slightly. The increased C–O contribution suggests the enrichment of oxygen-containing surface groups after Fe modification and is consistent with the possible formation of Fe-associated interfacial oxygen structures, such as Fe–O–C linkages. Note that the C 1s peak-fitting results in Figure 2c and Figure 5a were obtained from two separate fitting batches with slightly adjusted fitting intervals; the absolute values therefore differ slightly, while the relative trends are identical. In the N 1s spectra (Figure 5c,d), pyridinic N decreased slightly from 31.4% to 28.5%, whereas oxidized N increased from 1.4% to 4.4%, indicating that Fe modification and thermal treatment modified the local nitrogen configuration and promoted partial oxidation of N-containing functionalities. The O 1s spectra (Figure 5e,f) were dominated by hydroxyl/adsorbed oxygen species, further supporting the increase in surface oxygen functionalities after Fe loading.

The Fe 2p spectra confirmed changes in iron speciation after modification (Figure 5g,h). Compared with SBC, Fe@SBC exhibited a higher proportion of Fe^2+^ (60.7% and 80.8%) and a lower proportion of Fe^3+^ (29.8% and 12.6%) (Table S3), which may be associated with carbothermal reduction in Fe species during pyrolysis under an inert atmosphere. The carbon matrix likely facilitated the preservation of part of the reduced Fe species. Such coexistence of Fe^2+^ and Fe^3+^ is expected to favor redox cycling and thereby promote PMS activation. In addition, the interaction between Fe species and the carbon matrix may facilitate interfacial electron transfer, contributing to the enhanced catalytic performance of Fe@SBC.

XRD results corroborated the successful introduction of iron species (Figure 5i,j). SBC showed a broad diffraction peak at approximately 20.85°, characteristic of disordered or poorly graphitized carbon, and a sharp peak near 26.63°, which can be assigned to graphitic carbon or mineral phases such as quartz. After Fe modification, Fe@SBC retained these main carbon-related features, while weak additional diffraction peaks appeared in the range of 30–65°, suggesting the presence of crystalline iron oxide phases, possibly Fe_3_O_4_ or γ-Fe_2_O_3_. These results indicate that iron species were successfully incorporated without destroying the basic carbon framework of the sludge-derived biochar.

The FTIR spectra in Figure S2 also showed obvious differences between SBC and Fe@SBC. A new Fe-O peak emerged in Fe@SBC, verifying successful iron loading. The strengthened signals of O-H and C-O bands indicate an increase in oxygen-containing groups after modification. Together with the XPS results, these findings suggest strengthened interactions between Fe species and the oxygenated carbon matrix, which are favorable for PMS activation.

Nitrogen adsorption–desorption analysis showed that both SBC and Fe@SBC exhibited type IV isotherms with H4 hysteresis loops, characteristic of mesoporous materials (Figure 5k). Fe@SBC displayed a higher adsorption capacity across the relative pressure range, reflecting improved pore development after Fe modification. Quantitatively, the BET specific surface area increased from 50.93 to 66.54 m^2^·g^−1^, while the total pore volume increased to 0.309 cm^3^·g^−1^ and the average pore diameter increased from 6.68 to 18.58 nm (Table S4). The much larger increase in pore volume than in surface area suggests that Fe incorporation promoted pore expansion and improved structural openness, rather than simply generating additional micropores. Such hierarchical porosity is advantageous for both pollutant enrichment and mass transfer, facilitating the diffusion of MO, PMS, and reactive intermediates to and from the active sites.

The CV curves of SBC and Fe@SBC (Figure S3) exhibited quasi-rectangular shapes without obvious redox peaks, indicating that the electrochemical response was dominated by electrical double-layer capacitance. Compared with SBC, Fe@SBC showed a higher current response and a larger enclosed area, suggesting enhanced interfacial charge storage and charge-transfer capability after Fe modification. This improvement is likely related to the increased surface area, modified surface chemistry, and stronger interaction between Fe species and the carbon matrix. Although no pronounced faradaic peaks were observed, the improved electrochemical response is consistent with the superior PMS activation performance of Fe@SBC.

### 3.3. Effects of Operational Parameters on Catalytic Performance of the Fe@SBC/PMS System

#### 3.3.1. PMS Dosage

**Figure 6:**
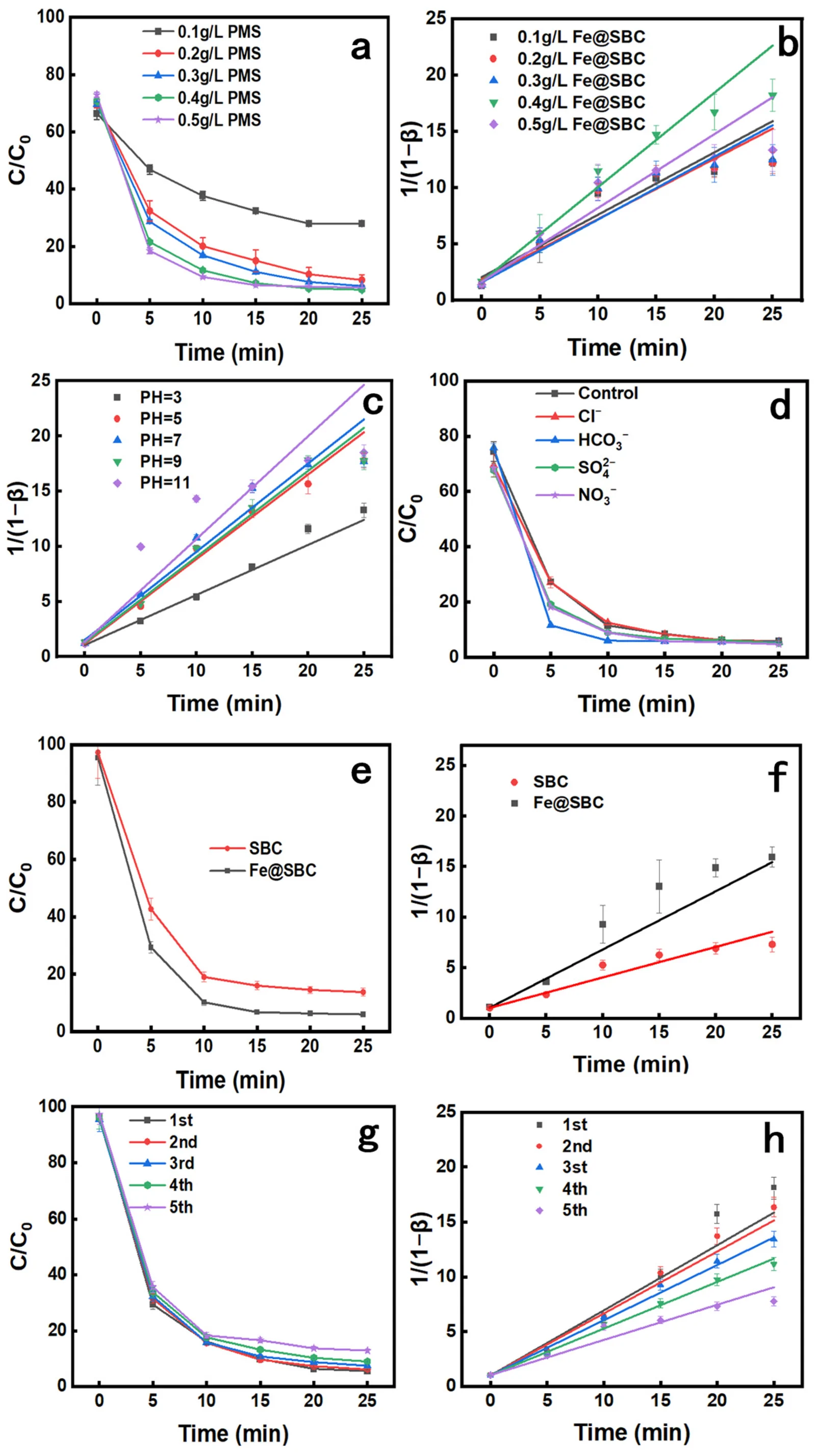
Effects of different reaction conditions on methyl orange degradation efficiency in the Fe@SBC/PMS system (**a**–**d**), comparison of MO degradation efficiency by SBC and Fe@SBC activated PMS (**e**,**f**), and cyclic performance of Fe@SBC (**g**,**h**).

The PMS concentration exerted a substantial influence on MO removal efficiency (Figure 6a). As the PMS dosage increased from 0.1 to 0.4 g·L^−1^, the degradation efficiency rose markedly from 71.9 ± 0.1% to 94.2 ± 0.2%. This enhancement is attributable to the increased availability of PMS molecules as precursor pools for ROS generation. A higher oxidant concentration favors the probability of collision with active sites on the Fe@SBC surface, thereby accelerating the oxidative decomposition of the target contaminant. However, further increasing the PMS concentration to 0.5 g·L^−1^ yielded only a marginal improvement of 0.3%, with the removal efficiency reaching 95.5 ± 0.2%. This plateau suggests that the system approaches saturation in oxidant utilization. The phenomenon can be rationalized by the occurrence of non-productive side reactions at elevated PMS levels, wherein excess oxidant scavenges the generated radicals through pathways such as SO_4_^•−^ + HSO_5_^−^ → SO_5_^•−^ + HSO_4_^−^ and ^•^OH + HSO_5_^−^ → SO_5_^•−^ + H_2_O, effectively diminishing the steady-state concentration of active species available for pollutant attack [32]. Based on both efficiency and economic considerations, 0.4 g·L^−1^ was selected as the optimal PMS dosage.

#### 3.3.2. Fe@SBC Dosage

The influence of catalyst loading on MO degradation was examined over the range of 0.1–0.5 g·L^−1^ (Figure 6b). The removal efficiency exhibited a clear increasing trend from 91.5 ± 0.3% to 95.0 ± 0.2% as the Fe@SBC dosage was raised from 0.1 to 0.4 g·L^−1^. This enhancement is ascribed to the increased availability of active sites, including surface Fe species, oxygen-containing functional groups, and carbon defects, which collectively participate in PMS activation and ROS generation [33]. Nevertheless, a further increase in catalyst dosage to 0.5 g·L^−1^ led to a slight decline in removal efficiency (1.6%), indicating that the system had approached an optimal loading threshold. This trend likely results from the interplay between enhanced active-site availability and the adverse effects of excessive solid concentration. At elevated dosages, catalyst agglomeration can reduce the exposed surface area and hinder mass transfer, while surplus catalyst may also facilitate non-productive scavenging of reactive species or radical recombination on the catalyst surface, thereby lowering the utilization efficiency of reactive species [34]. Consequently, the optimal Fe@SBC dosage was determined to be 0.4 g·L^−1^.

#### 3.3.3. Initial pH

The pH dependence of the Fe@SBC/PMS system was evaluated across a broad range from 3 to 11 (Figure 6c). Notably, MO removal remained highly efficient at all tested pH values, with final efficiencies ranging from 93.0 ± 0.4% to 95.2 ± 0.2%. The relatively minor variation underscores the exceptional pH adaptability of this system. Among the tested conditions, the most rapid initial degradation was observed at pH 11, suggesting that alkaline conditions favor the early-stage generation of reactive species. This phenomenon may be related to the base-induced activation of PMS or the accelerated transformation of surface iron species under alkaline environments. Nevertheless, the overall stability across pH 3–11 is particularly noteworthy, as it indicates the suitability of the system for treating wastewater with fluctuating pH conditions without the need for pre-adjustment. The robust pH tolerance is a distinct advantage over conventional Fenton-based processes, which are severely constrained by narrow operational pH windows (2–4) [35].

#### 3.3.4. Coexisting Anions

The influence of common inorganic anions (Cl^−^, NO_3_^−^, SO_4_^2−^, and HCO_3_^−^) on MO degradation was assessed at a concentration of 10 mmol·L^−1^ (Figure 6d). Remarkably, the presence of Cl^−^, NO_3_^−^, and SO_4_^2−^ resulted in minimal suppression of degradation, with all systems achieving MO removal exceeding 94% at 25 min. This resistance to anionic interference underscores the robust nature of the Fe@SBC/PMS system, likely attributable to the predominance of the non-radical pathway, which is largely unaffected by typical inorganic anions. Interestingly, the HCO_3_^−^-containing system exhibited a slightly promoted degradation (95.5 ± 0.2%) compared with the control, particularly during the initial 10 min of reaction. This observation can be rationalized by the conversion of HCO_3_^−^ to CO_3_^•−^ (*E*^0^ = 1.78 V) via reaction with ^•^OH; CO_3_^•−^ retains moderate oxidizing capacity and can participate in the oxidative transformation of organic contaminants. Over prolonged reaction periods, however, the buffering effect of HCO_3_^−^/CO_3_^2−^ may gradually elevate the solution pH, causing a minor retardation. Collectively, these results confirm the excellent anti-interference capability of the Fe@SBC/PMS system toward typical coexisting anions, demonstrating its practical viability for treating complex real wastewater matrices.

### 3.4. Degradation Performance of Fe@SBC at Optimized Conditions

Under the optimized conditions (0.4 g·L^−1^ PMS, 0.4 g·L^−1^ Fe@SBC, ambient pH), the catalytic performance of SBC and Fe@SBC was comparatively evaluated for MO degradation (Figure 6e,f). Both materials exhibited appreciable PMS activation capability; however, Fe@SBC consistently outperformed its unmodified counterpart throughout the entire reaction course. At 25 min, the MO removal efficiency reached 95.2 ± 0.2% for Fe@SBC, significantly surpassing the 86.0 ± 0.6% achieved by SBC. This enhancement of approximately 9.2 percentage points unequivocally demonstrates the critical role of iron modification in elevating the catalytic activity of sludge biochar. The approximately 9.2% (*p* < 0.05) improvement clearly demonstrates that iron modification substantially enhances the catalytic activity of sludge biochar. This superior performance can be attributed to the increased specific surface area and pore volume, which facilitate pollutant diffusion and adsorption; the abundant Fe^2+^ species, which serve as active sites for PMS activation; the Fe–O–C interfacial structure, which promotes electron transfer; and the enriched oxygen-containing functional groups and graphitic nitrogen, which may contribute to non-radical activation pathways [22]. Overall, these results highlight iron modification as an effective strategy for converting sludge biochar into a more efficient catalyst for advanced oxidation processes.

### 3.5. TOC Removal Capacity

The TOC elimination curves in Figure S4 reflect the mineralization capacity of two systems. The SBC/PMS group only achieved slight TOC reduction, while Fe@SBC/PMS exhibited remarkable mineralization efficiency, with nearly 40% total organic carbon removed within 300 min. This indicates iron modification greatly promoted thorough mineralization of MO instead of merely decolorization.

### 3.6. Reusability and Stability of Fe@SBC

The reusability and stability of Fe@SBC were evaluated over five consecutive degradation cycles, with the used catalyst regenerated by sequential washing with dilute HCl and ethanol followed by drying at 80 °C. As illustrated in Figure 6g, the Fe@SBC/PMS system maintained high catalytic performance throughout the recycling tests. The MO removal efficiencies after 25 min were 94.4 ± 0.3%, 93.0 ± 0.4%, 91.5 ± 0.5%, 90.0 ± 0.6%, and 88.0 ± 0.8% from the first to fifth cycles, respectively. The overall decrease of only 6.4% after five runs indicates good reusability of the catalyst.

Kinetic analysis based on the pseudo-first-order model (Figure 6h) showed that the apparent rate constant decreased from 0.5940 min^−1^ in the first cycle to 0.3203 min^−1^ in the fifth cycle, corresponding to a 46.1% reduction. This result suggests gradual deactivation of the most accessible or highly active surface sites, although the catalyst still retained substantial activity. The decline in catalytic performance may be attributed to partial leaching of surface Fe species during acid washing, accumulation of refractory intermediates on the catalyst surface, and oxidative deterioration during repeated use. Despite this kinetic attenuation, Fe@SBC still achieved more than 88% MO removal within 25 min after five cycles, confirming its favorable stability and practical reusability. Further improvement in cycling performance may be achieved by optimizing the regeneration procedure or enhancing the immobilization of Fe species on the carbon matrix.

### 3.7. Degradation Products and Pathway Analysis

**Figure 7:**
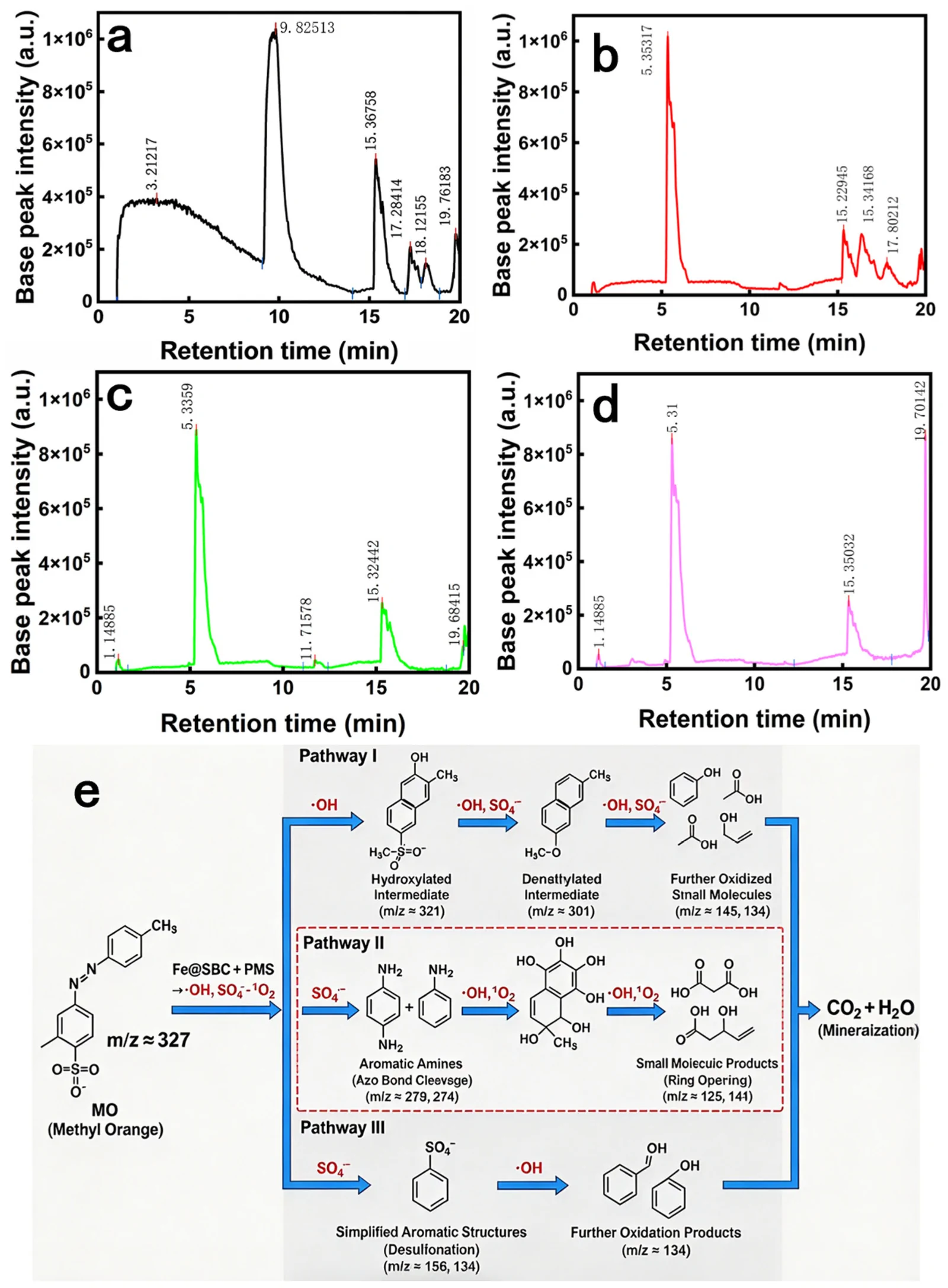
LC-MS chromatograms of methyl orange at different reaction times in the Fe@SBC/PMS system: (**a**) 0 min, (**b**) 60 min, (**c**) 180 min, (**d**) 300 min; (**e**) degradation pathway of methyl orange.

LC-MS analysis was conducted on samples collected at 0, 60, 180, and 300 min to investigate the transformation of MO during Fe@SBC/PMS treatment, and the corresponding chromatograms are shown in Figure 7. At 0 min (Figure 7a), the chromatogram was dominated by the parent compound of MO, with the [M-H]^−^ parent ion at *m*/*z* = 327 in negative ionization mode. After 60 min (Figure 7b), several new signals appeared, which can be tentatively assigned to oxidized and fragmented intermediates, including a hydroxylated product at *m*/*z* = 321 and species at *m*/*z* = 279 and 274 that were likely associated with azo bond cleavage and partial substitution of the aromatic structure. These changes suggest that hydroxylation and cleavage of the azo linkage occurred during the early stage of oxidation. After 180 min (Figure 7c), the signal of the parent compound had largely disappeared, while intermediates with lower *m*/*z* values, such as 279 and 125, became more evident, indicating further transformation of the initial aromatic fragments. At 300 min (Figure 7d), additional low molecular weight products, including signals at *m*/*z* = 141 and 125, were detected, suggesting continued oxidation, dealkylation, and fragmentation of aromatic intermediates into smaller molecules. These results indicate that MO degradation in the Fe@SBC/PMS system proceeded through successive oxidation and fragmentation steps rather than a single-step conversion.

Based on these observations, a possible degradation scheme is proposed in Figure 7e. One route (Pathway I) involves initial hydroxylation of MO, followed by further oxidation and demethylation to generate lower molecular weight intermediates. Another likely route (Pathway II) is cleavage of the azo bond, producing aromatic fragments that subsequently undergo hydroxylation, dealkylation, and further oxidation. Desulfonation may also occur during the degradation process, leading to structurally simplified intermediates that are more susceptible to subsequent oxidation. These intermediates are then converted into smaller organic molecules through continued oxidative transformation, and partial mineralization may occur with prolonged reaction. This stepwise and multi-route degradation behavior is consistent with the high oxidation capacity of the Fe@SBC/PMS system.

### 3.8. Mechanism Analysis of MO Degradation by Fe@SBC-Activated PMS

#### 3.8.1. Identification of Dominant Reactive Species via Quenching Experiments

To identify the specific reactive oxygen species responsible for MO degradation and to quantify their relative contributions, quenching experiments were conducted using methanol (MeOH, quencher for both SO_4_^•−^ and ^•^OH), tert-butanol (TBA, quencher for ^•^OH), and L-histidine (quencher for ^1^O_2_).

**Figure 8:**
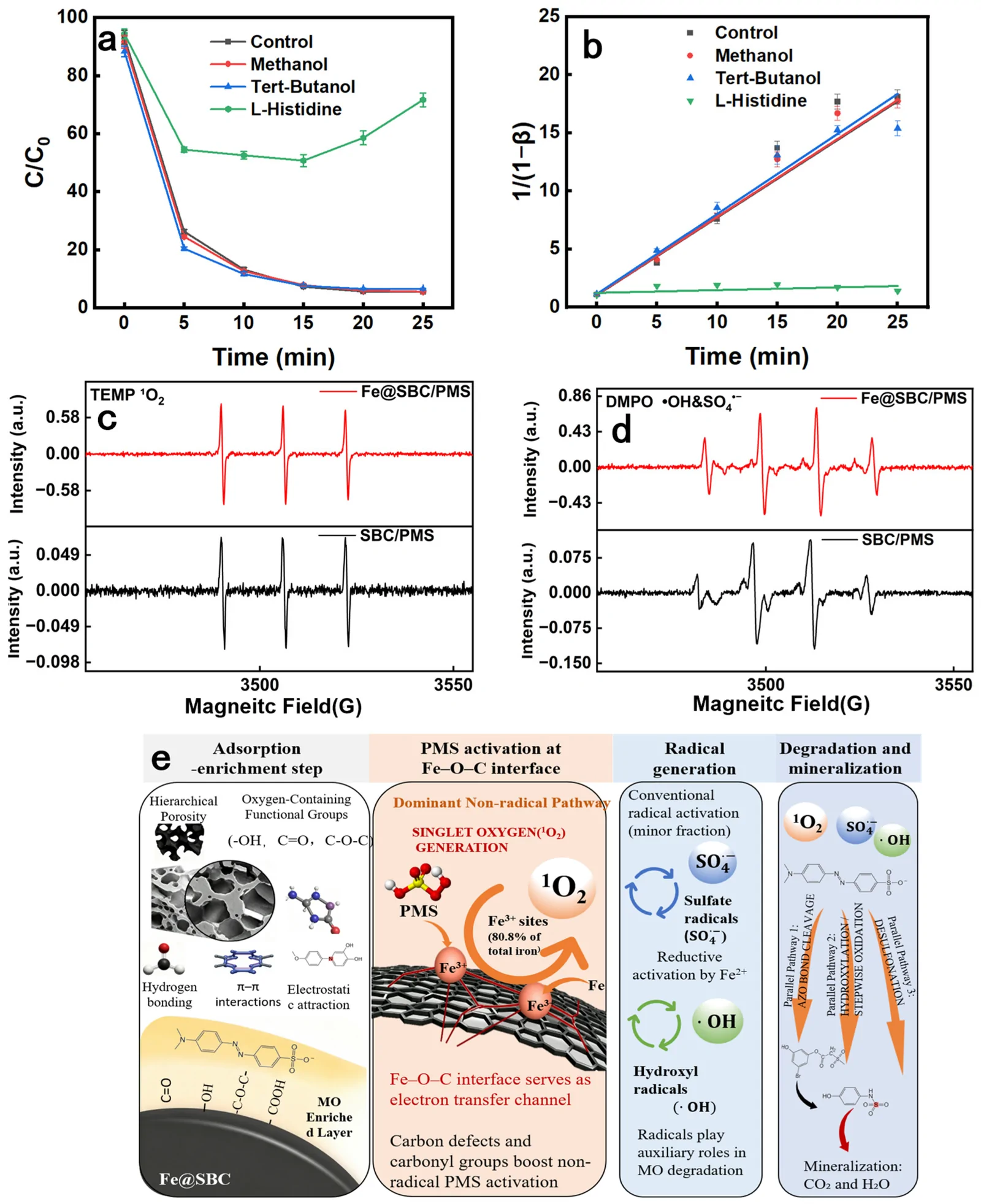
(**a**,**b**) Effects of different quenchers on MO degradation in the Fe@SBC/PMS system; (**c**,**d**) EPR spectra of reactive oxygen species in the system; (**e**) proposed mechanism scheme for MO degradation in the Fe@SBC/PMS system.

The addition of MeOH and TBA caused only marginal inhibition, with MO removal efficiencies of 95.0 ± 0.3% and 94.0 ± 0.3%, respectively, compared with 95.2 ± 0.2% for the control in the absence of inhibitor (Figure 8a). These results indicate that SO_4_^•−^ and ^•^OH made only a limited contribution to MO degradation under the tested conditions. In contrast, the addition of L-histidine drastically suppressed the reaction, reducing the removal efficiency to 48.0 ± 2.0%. Consistently, the apparent rate constant decreased from 0.5940 min^−1^ in the control to 0.1023 min^−1^ after L-histidine addition, corresponding to an 82.8% reduction (Figure 8b). These results suggest that ^1^O_2_ was the dominant reactive species in the Fe@SBC/PMS system, whereas radical species played a secondary role.

#### 3.8.2. Direct Detection of Reactive Species by EPR Spectroscopy

To further verify the nature of the reactive oxygen species, EPR spectroscopy was conducted using DMPO and TEMP as spin-trapping agents. As shown in Figure 8c,d, both Fe@SBC/PMS and SBC/PMS generated characteristic DMPO adduct signals assignable to ^•^OH and SO_4_^•−^, confirming the formation of radical species in both systems. The signal intensity was markedly higher in the Fe@SBC/PMS system, indicating that iron modification enhanced PMS activation and promoted radical generation. In addition, a typical three-line TEMP signal attributed to ^1^O_2_ was observed in both systems, and its intensity was also significantly stronger for Fe@SBC/PMS than for SBC/PMS. Combined with the quenching results, these findings demonstrate that both radical and non-radical pathways coexisted in the Fe@SBC/PMS system, while ^1^O_2_ was the predominant species responsible for MO degradation.

#### 3.8.3. Mechanistic Rationale for ^1^O_2_-Dominated Non-Radical Pathway

The predominance of ^1^O_2_ can be rationalized by the physicochemical properties of Fe@SBC. XPS analysis showed that Fe species on the catalyst surface were dominated by Fe^2+^, which may facilitate PMS adsorption and electron redistribution [22], thereby favoring non-radical oxidation pathways. Meanwhile, the coexistence of Fe^3+^/Fe^2+^ can sustain continuous PMS activation through surface redox cycling. The Fe–O–C interfacial structure formed between iron species and the carbon matrix may further accelerate interfacial electron transfer, which is beneficial for PMS activation. In addition, the enriched oxygen-containing functional groups and defective carbon structure can serve as adsorption and electron-transfer sites, promoting the formation of non-radical oxidizing species. The contribution of graphitic nitrogen may also be important, as it can modulate the electronic structure of adjacent carbon atoms and enhance surface electron mobility. Since MO contains electron-rich functional groups such as azo bonds and aromatic rings, it is susceptible to attack by ^1^O_2_ [36], which helps explain the high degradation efficiency observed in this system.

#### 3.8.4. Mechanism of Methyl Orange Degradation in Fe@SBC/PMS System

Based on the catalyst characterization and the results of quenching, EPR, and LC–MS analyses, a plausible mechanism for MO degradation in the Fe@SBC/PMS system is proposed in Figure 8e. The process involves adsorption of MO on Fe@SBC, PMS activation on the catalyst surface, and subsequent oxidative degradation.

The hierarchical pore structure and surface oxygen-containing groups of Fe@SBC favor MO enrichment through pore filling, hydrogen bonding, π–π interaction, and electrostatic attraction, thus increasing the accessibility of MO to surface reactive sites. After adsorption, PMS is activated mainly at Fe–O–C interfacial sites. The Fe-rich surface, particularly the dominant Fe^2+^ species, together with carbon defects and carbonyl groups, promotes electron transfer and facilitates the non-radical conversion of PMS to ^1^O_2_. Meanwhile, a minor fraction of PMS is activated through the Fe^2+^/Fe^3+^ redox cycle to produce SO_4_^•−^ and ^•^OH. Quenching and EPR results consistently indicate that both radical and non-radical pathways coexist, while ^1^O_2_ is the predominant reactive species.

The generated reactive oxygen species then attack MO through multiple transformation routes, including azo bond cleavage, hydroxylation or stepwise oxidation, and desulfonation. These reactions produce smaller intermediates, which are further oxidized into low-molecular-weight compounds and eventually partially mineralized. The superior catalytic performance of Fe@SBC can therefore be attributed to the synergistic effects of hierarchical porosity, Fe active sites, Fe–O–C interfacial structures, carbon defects, and oxygen-containing functional groups, which collectively enhance pollutant adsorption and PMS activation. Overall, the Fe@SBC/PMS system follows an adsorption-assisted and ^1^O_2_-dominated oxidation mechanism, with SO_4_^•−^ and ^•^OH making secondary contributions to MO degradation.

### 3.9. Comparative Analysis and Outlook

#### 3.9.1. Performance Comparison with Previous Fe-Modified Sludge Biochar

Table 1 compares the catalytic performance of Fe@SBC with reported Fe-modified sludge biochar/PMS systems. Existing catalysts have demonstrated good activity toward a wide range of pollutants, including azo dyes, antibiotics, and perfluorinated compounds, with PMS activation proceeding through either mixed radical/non-radical pathways or predominantly non-radical routes. For example, Mg-Fe co-modified sludge biochar achieved efficient removal of azo dyes and antibiotics through radical and non-radical synergy [27]. FeOCl one-pot loading sludge biochar exhibited a ^1^O_2_- and O_2_^•−^-dominated radical pathway for organic pollutant degradation [37]. Hydrothermal Fe-doped sludge biochar achieved 85.6% tetracycline degradation with low iron leaching [38]. In these systems, the enhanced catalytic performance has generally been attributed to the synergistic effects of Fe redox centers, defect-rich carbon structures, and oxygen- or nitrogen-containing functional groups.

Compared with these reported systems, Fe@SBC achieves faster dye decolorization (95.2 ± 0.2% within 25 min) with 38.0% TOC mineralization, wider pH tolerance (3–11), and better cycling stability (88.0% after five cycles). More importantly, while most reported systems involve mixed radical and non-radical pathways, our study quantitatively identifies ^1^O_2_ as the dominant species with ~80% contribution, with sulfate and hydroxyl radicals playing auxiliary roles. This non-radical dominance is achieved through systematic optimization of pyrolysis parameters (800 °C, 10 °C·min^−1^) and Fe–O–C interfacial construction, which direct PMS activation toward ^1^O_2_ generation rather than conventional radical chain reactions.

#### 3.9.2. Outlook for Further Research

Although Fe@SBC exhibited efficient catalytic activity, good pH tolerance, and satisfactory reusability, several issues should be addressed before practical application. First, its performance should be validated in real wastewater and continuous-flow systems, where coexisting salts, surfactants, and natural organic matter may influence PMS utilization and pollutant degradation. Second, long-term environmental safety requires further evaluation, particularly with respect to iron leaching, catalyst stability, and the toxicity of treated effluents and degradation intermediates. Third, further optimization of catalyst design, such as heteroatom doping or bimetallic modification, may enhance mineralization efficiency and broaden applicability to other refractory contaminants. Finally, techno-economic assessment and scalable recovery strategies should be considered to evaluate the feasibility of full-scale implementation. These efforts will help advance sludge-derived Fe-carbon catalysts from laboratory study toward practical wastewater treatment applications.

## 4. Conclusions

This work demonstrates that Fe@SBC is a cost-effective and sustainable catalyst for PMS activation, achieving 95.2 ± 0.2% MO removal within 25 min with excellent pH adaptability (3–11) and reusability (88 ± 0.8% after five cycles). Mechanistic studies identify ^1^O_2_ as the dominant reactive species (~80% contribution), with sulfate and hydroxyl radicals playing auxiliary roles, enabled by the synergy of Fe^2+^ species, Fe–O–C interfaces, and carbon defects. LC-MS reveals three parallel degradation pathways involving azo bond cleavage, hydroxylation, and desulfonation. These findings advance the understanding of non-radical mechanisms in iron–carbon systems and establish a viable strategy for integrating sludge valorization with dye wastewater treatment.

## Figures and Tables

**Table 1:** Performance comparison with authentic published Fe-biochar/PMS studies.

Catalyst Raw Material & Modification	Optimal Dosages	MO/Azo Dye Removal Rate	300 min TOC Mineralization	pH Range	Dominant ROS	Removal After 5 Cycles	Reference
Municipal sludge, FeCl_3_ impregnation co-pyrolysis	0.4 g·L^−1^ Fe@SBC, 0.4 g·L^−1^ PMS	95.2% (25 min)	38.00%	3–9, slight fluctuation	^1^O_2_ (~80% contribution)	88.00%	This work
-Fe co-modified sludge biochar	0.45 g·L^−1^ catalyst, 0.45 g PMS	90.4% (30 min)	24.70%	4–8	Coexistence of ^1^O_2_ & radicals	81.20%	[27]
Sewage sludge, FeOCl one-pot loading	0.5 g·L^−1^ catalyst, 0.5 g·L^−1^ PMS	87.0% (30 min)	19.20%	5–8, sharp drop at pH < 5	^•^OH + SO_4_^•−^ dominant	76.30%	[37]
Hydrothermal Fe-doped sludge carbon	0.6 g·L^−1^ catalyst, 0.6 g·L^−1^ PMS	85.6% (30 min)	16.40%	6–9, nearly inactive in acid	Free radicals are absolutely dominant	72.10%	[38]

## Data Availability

The original contributions presented in this study are included in the article/Supplementary Material. Further inquiries can be directed to the corresponding author.

## Supplementary Materials

The following supporting information can be downloaded at: https://www.mdpi.com/article/10.3390/nano16191233/s1; Figure S1: preparation flow chart of iron-modified sludge biochar; Figure S2: Fourier-transform infrared spectra of SBC and Fe@SBC; Figure S3: cyclic voltammetry curves of SBC and Fe@SBC; Figure S4: comparison of TOC removal performance between SBC/PMS and Fe@SBC/PMS systems; Table S1: summary of experimental design for MO degradation in the biochar/PMS; Table S2: relative atomic percentages of C, N, O and Fe determined by EDS for washed raw sludge, SBC and Fe@SBC; Table S3: quantitative proportion of Fe^2+^ and Fe^3+^ after fitting Fe 2p high-resolution XPS spectra; Table S4: specific surface area, total pore volume, and average pore size of SBC and FM@SBC. Text S1: cThe following reagents were used as received without further purification. MO and PMS (42.0–46.0% 2KHSO5·KHSO4·K2SO4) were purchased from Beijing InnoChem Science & Technology Co., Ltd. Ferric chloride hexahydrate (FeCl3·6H2O, ≥99.0%) was obtained from Sinopharm Chemical Reagent Co., Ltd. Methanol (MeOH) and benzoic acid (BA) were obtained from Beijing Mairuida Technology Co., Ltd. L-histidine (L-His) was obtained from J&K Scientific Ltd. P-benzoquinone (BQ, ≥99.0%) was purchased from Shanghai Macklin Biochemical Co., Ltd. Additional chemicals included KOH (96%, Beijing Ruixiang Nengyuan) and H2SO4 (98%, Beijing Yili Fine Chemicals). All compounds were 99.0% purity unless otherwise specified; Text S2: Electron paramagnetic resonance (EPR) spectra were collected with Bruker Emxplus X-band EPR spectrometer, (Karlsruhe, Germany), it was used to qualitatively capture reactive oxygen species (ROS). DMPO (5,5-dimethyl-1-pyrrolidine-N-oxide) and TEMP (4-amino-2,2,6,6-tetramethylpiperidine) were used as the spin-trapping agents in the EPR experiments. DMPO were used as a spin trap to detect SO_4_•^−^ and •OH, TEMP was used to capture singlet oxygen (^1^O_2_). After 1 min of catalytic reaction, 1 mL mixed solution was taken, mixed with 10 μL spin-trapping agent rapidly, transferred into capillary tube immediately for EPR signal scanning. The characteristic peak shape and signal intensity of adducts (DMPO–OH, DMPO–SO_4_^−^, TEMPO–^1^O_2_) were compared to distinguish ROS types and relative yield. For the EPR detection of singlet oxygen (^1^O_2_), a 1:1 (*v*/*v*) methanol/water mixture was used as the electrolyte; Text S3: Intermediates analysis. The intermediates from MO degradation were identified by high-resolution liquid chromatography-mass spectrometry (LC-QTOF-MS, Agilent 1290 Infinity II/6550, USA) operated in both positive and negative ionization modes. The chromatographic separation was completed on a system equipped with a C18 reversed-phase column (2.1 × 100 mm, 1.7 μm). The mobile phase consisted of (A) 0.1% formic acid in water and (B) acetonitrile. The gradient elution program was set to: 0–2 min, 5% B; 2–10 min, 5–50% B; 10–15 min, 50–95% B; 15–18 min, 95% B; 18–20 min, 95–5% B. The flow rate was 0.3 mL/min and the injection volume was 5 μL. Electrospray ionization (ESI) source was used for mass spectrometry detection, and scanning was carried out in positive and negative ion mode to improve the detection sensitivity and coverage of different types of intermediates.
