# Supplementary material for: Degradation Performance and Mechanism of Methyl Orange by Iron-Modified Sludge Biochar-Activated Peroxymonosulfate
Source: Nanomaterials (Basel). 2026 Sep 30;16(19):1233. doi: 10.3390/nano16191233 (PMC13667204; doi:10.3390/nano16191233)
Supplement: Supplementary file 1 [file nanomaterials-16-01233-s001.zip › nanomaterials-4521138-supplementary.pdf]

Supplementary Material

Degradation Performance and Mechanism of Methyl Orange by Iron-Modified Sludge Biochar Activated Peroxymonosulfate

Meng-Yuan Li[1], Jiang-Li Yu[1], Mei-Qi Ren[1], Tao Hou[1], Dan Cui[1]*

[1] National Engineering Laboratory for Advanced Municipal Wastewater Treatment and Reuse Technology, College of Environmental Science and Engineering, Beijing University of Technology, Beijing 100124, PR China

* Corresponding author

E-mail: cuidanflying@bjut.edu.cn (D. Cui)

Text S1

The following reagents were used as received without further purification. MO and PMS (42.0-46.0% $2KHSO_5 \cdot KHSO_4 \cdot K_2SO_4$) were purchased from Beijing InnoChem Science & Technology Co., Ltd. Ferric chloride hexahydrate ($FeCl_3 \cdot 6H_2O$, ≥99.0%) was obtained from Sinopharm Chemical Reagent Co., Ltd. Methanol (MeOH) and benzoic acid (BA) were obtained from Beijing Mairuida Technology Co., Ltd. L-histidine (L-His) was obtained from J&K Scientific Ltd. P-benzoquinone (BQ, ≥99.0%) was purchased from Shanghai Macklin Biochemical Co., Ltd. Additional chemicals included KOH (96%, Beijing Ruixiang Nengyuan) and H_2SO_4 (98%, Beijing Yili Fine Chemicals). All compounds were 99.0% purity unless otherwise specified.

Text S2

Electron paramagnetic resonance (EPR) spectra were collected with Bruker Emxplus X-band EPR spectrometer, (Karlsruhe, Germany), it was used to qualitatively capture reactive oxygen species (ROS). DMPO (5,5-dimethyl-1-pyrrolidine-N-oxide) and TEMP (4-amino-2,2,6,6-tetramethylpiperidine) were used as the spin-trapping agents in the EPR experiments. DMPO were used as a spin trap to detect $SO_4^{\bullet-}$ and •OH, TEMP was used to capture singlet oxygen ($^1O_2$). After 1 min of catalytic reaction, 1 mL mixed solution was taken, mixed with 10 μL spin-trapping agent rapidly, transferred into capillary tube immediately for EPR signal scanning. The characteristic peak shape and signal intensity of adducts (DMPO–OH, DMPO–SO_4^-, TEMPO–$^1O_2$) were compared to distinguish ROS types and relative yield. For the EPR detection of singlet oxygen ($^1O_2$), a 1:1 (v/v) methanol/water mixture was used as the electrolyte.

Text S3

Intermediates analysis. The intermediates from MO degradation were identified by high-resolution liquid chromatography-mass spectrometry (LC-QTOF-MS, Agilent 1290 Infinity II/6550, USA) operated in both positive and negative ionization modes. The chromatographic separation was completed on a system equipped with a C18 reversed-phase column (2.1 × 100mm,1.7 μm). The mobile phase consisted of (A)0.1% formic acid in water and (B) acetonitrile. The gradient elution program was set to: 0-2 min,5% B;2-10 min,5%-50% B;10-15 min,50%-95% B;15-18 min,95% B;18-20 min,95%-5% B. The flow rate was 0.3 mL/min and the injection volume was 5 μL. Electrospray ionization (ESI) source was used for mass spectrometry detection, and scanning was carried out in positive and negative ion mode to improve the detection sensitivity and coverage of different types of intermediates.

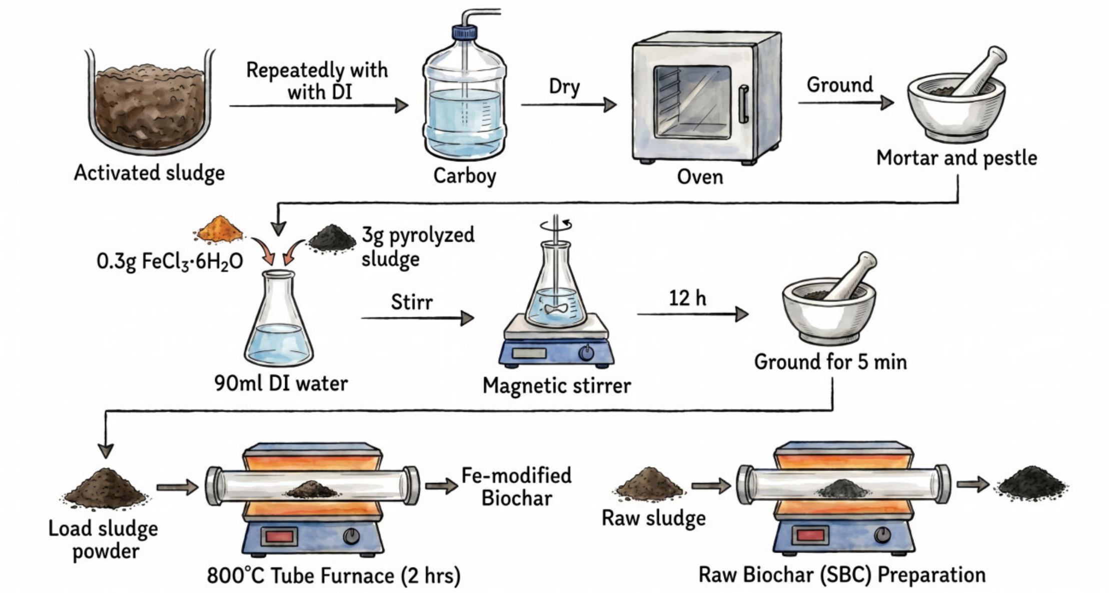

Figure S1. Preparation flow chart of iron-modified sludge biochar

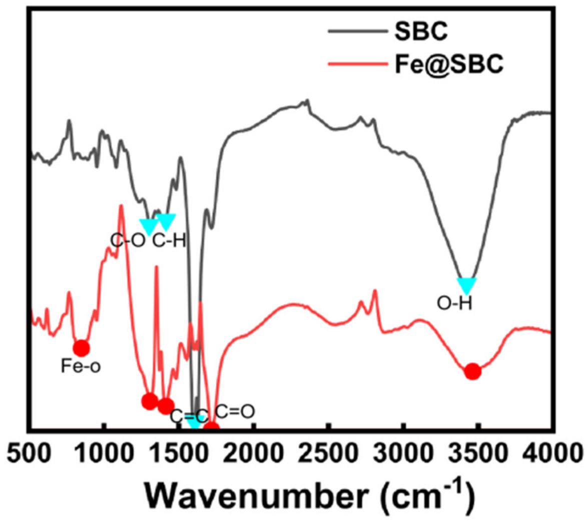

Figure S2. Fourier transform infrared spectra of SBC and Fe@SBC

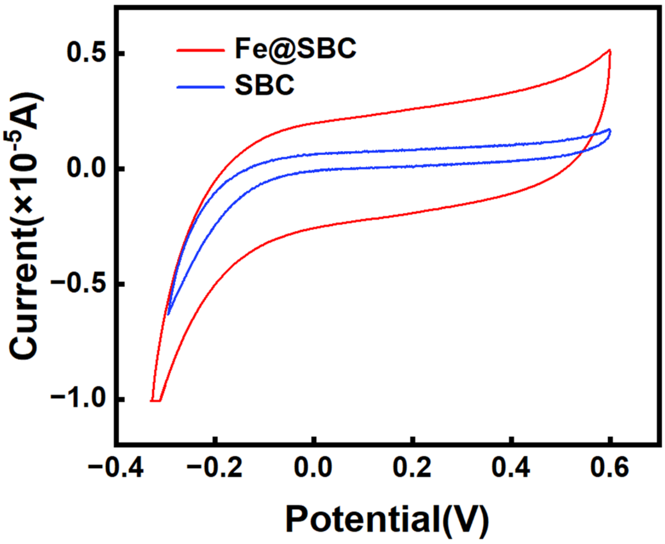

Figure S3. Cyclic voltammetry curves of SBC and Fe@SBC

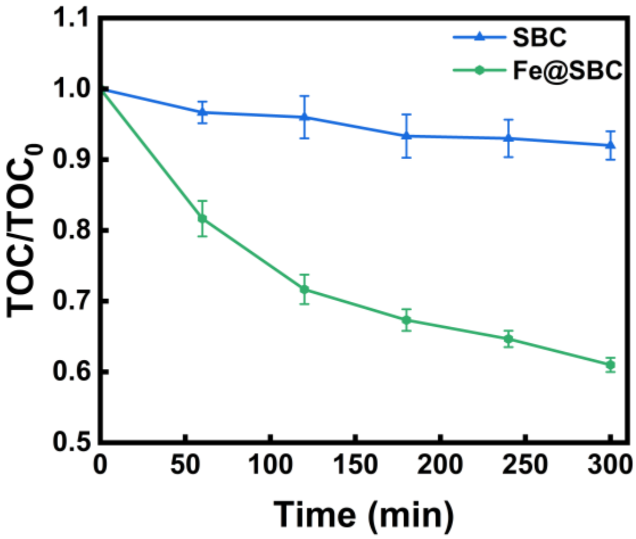

Figure S4. Comparison of TOC removal performance between SBC/PMS and Fe@SBC/PMS systems

Table S1 Summary of experimental design for MO degradation in the biochar/PMS

Category	Parameter	Control Group (SBC System)	Experimental Group (Fe@SBC System)
Core Components	Catalyst	Unmodified Biochar (SBC)	Modified Biochar (Fe@SBC)
	Oxidant	PMS (added at 30 min)	PMS (added at 30 min)
Reaction Stages	0–30 min	Adsorption equilibrium	Adsorption equilibrium
	30–55 min	Catalytic oxidation	Catalytic oxidation
Operational Variables	Catalyst Dosage	0.40 g/L (Fixed)	0.10, 0.20, 0.30, 0.40, 0.50 g/L
	PMS Concentration	0.40 g/L (Fixed)	0.10, 0.20, 0.30, 0.40, 0.50 g/L
	Initial pH	8.2 (Unadjusted)	3.0, 5.0, 7.0, 9.0, 11.0
	Coexisting Anions	None	Cl^-, NO_3^-, HCO_3^-, and SO_4^{2-} (10 mM)
Fixed Conditions	Temperature & Agitation	25 °C, 200 rpm	25 °C, 200 rpm
Evaluation Metrics	Performance & Kinetics	Total removal efficiency; k_{obs}	Total removal efficiency; k_{obs}

Table S2 Relative atomic percentages of C, N, O and Fe determined by EDS for washed raw sludge, SBC and Fe@SBC

Element	Washed raw sludge (at.%)	SBC (at.%)	Fe@SBC (at.%)
C	26.69	46.92	37.70
N	6.83	2.39	0.53
O	65.23	47.79	55.43
Fe	1.25	2.89	6.34

Note: The atomic percentages were normalized to the total content of C, N, O, and Fe and are intended for comparative analysis of the major elements associated with pyrolysis and Fe modification.

Table S3 Quantitative proportion of Fe^{2+} and Fe^{3+} after fitting Fe 2p high-resolution XPS spectra

Iron valence fraction	SBC	Fe@SBC	Change
Fe^{2+}	60.7%	80.8%	Remarkable increase
Fe^{3+}	29.8%	12.6%	Obvious decrease

Table S4 Specific surface area, total pore volume, and average pore size of SBC and Fe@SBC

Sludge biochar	Specific surface area ($m^2 \cdot g^{-1}$)	Total pore volume ($cm^3 \cdot g^{-1}$)	Average pore diameter (nm)
SBC	50.93	0.085	6.68
Fe@SBC	66.54	0.309	18.58
